# Antimicrobial resistance and genomic characterization of *Salmonella enterica* isolates from chicken meat

**DOI:** 10.3389/fmicb.2023.1104164

**Published:** 2023-03-30

**Authors:** Khaloud O. Alzahrani, Fahad M. AL-Reshoodi, Elaf A. Alshdokhi, Ashwaq S. Alhamed, Meshari A. Al Hadlaq, Mohammed I. Mujallad, Lenah E. Mukhtar, Amani T. Alsufyani, Abdullah A. Alajlan, Malfi S. Al Rashidy, Mashan J. Al Dawsari, Saleh I. Al-Akeel, Meshari H. AL-Harthi, Abdulaziz M. Al Manee, Majed F. Alghoribi, Suliman M. Alajel

**Affiliations:** ^1^Molecular Biology Division, Reference Laboratory for Microbiology, Executive Department of Reference Laboratories, Research and Laboratories Sector, Saudi Food and Drug Authority (SFDA), Riyadh, Saudi Arabia; ^2^Antimicrobial Resistance Division, Reference Laboratory for Microbiology, Executive Department of Reference Laboratories, Research and Laboratories Sector, Saudi Food and Drug Authority (SFDA), Riyadh, Saudi Arabia; ^3^Microbial Identification Division, Reference Laboratory for Microbiology, Executive Department of Reference Laboratories, Research and Laboratories Sector, Saudi Food and Drug Authority (SFDA), Riyadh, Saudi Arabia; ^4^Microbiology Section, Food Laboratory, Laboratories Executive Department, Saudi Food and Drug Authority (SFDA), Riyadh, Saudi Arabia; ^5^Microbial Hazards Division, Risk Assessment Department, Executive Department of Monitoring and Risk Assessment, Saudi Food and Drug Authority (SFDA), Riyadh, Saudi Arabia; ^6^Biology Department, Faculty of Sciences, King Abdulaziz University, Jeddah, Saudi Arabia; ^7^Infectious Diseases Research Department, King Abdullah International Medical Research Center (KAIMRC), Riyadh, Saudi Arabia; ^8^Department of Basic Science, College of Science and Health Professions, King Saud Bin Abdulaziz University for Health Sciences (KSAU), Riyadh, Saudi Arabia; ^9^Reference Laboratory for Microbiology, Executive Department of Reference Laboratories, Research and Laboratories Sector, Saudi Food and Drug Authority (SFDA), Riyadh, Saudi Arabia

**Keywords:** *Salmonella*, whole genome sequencing, molecular epidemiology, molecular typing, virulence factors, antimicrobial resistant genes (ARG), phylogenetic analysis

## Abstract

This study investigated genotypic and phenotypic antimicrobial resistance profiles, phylogenic relatedness, plasmid and virulence composition of 39 *Salmonella enterica* strains isolated from chicken meat samples using whole genome sequencing (WGS) technology. Four distinct serotypes were identified; *Salmonella* Minnesota (16/39, 41%), *Salmonella* Infantis (13/39, 33.3%), *Salmonella* Enteritidis (9/39, 23.1%), and one isolate was detected for *Salmonella* Kentucky (1/39, 2.6%), with sequence types (STs) as followed: ST548, ST32, ST11, and ST198, respectively. Phenotypic resistance to tetracycline (91.2%), ampicillin (82.4%), sulfisoxazole (64.7%), and nalidixic acid (61.6%) was the most observed. Resistome analysis revealed the presence of resistance genes to aminoglycosides, β-lactamase, sulfonamides, trimethoprim, phenicol, lincosamide, macrolides, and tetracyclines. Plasmidome showed the presence of eight incompatibility groups, including IncA/C2, IncFIB(K)_1_Kpn3, Col440I_1, IncR, IncX1, IncI1_1_Alpha, IncFIB(S)/IncFII(S), IncHI2/IncHI2A, IncX2 and ColpVC plasmids across the 39 genomes. Three resistance genes, *sul2, tetA* and *bla_CMY-2,_* were predicted to be located on IncA/C2 plasmid in *S.* Minnesota isolates, whereas all *S.* Infantis isolates were positive to IncFIB(K)_1_Kpn3 plasmid that carries *bla*_CTX-M-65_ gene. Eleven *Salmonella* pathogenicity islands and up to 131 stress and/or virulence genes were identified in the evaluated genomes. Phylogenetic analysis showed four phylogroups that were consistent with the identified ST profiles with a high level of inter-diversity between isolates. This is the first genomic characterization of *Salmonella* isolates from retail chicken meat in Saudi Arabia using WGS technology. The availability of *Salmonella* genomes from multiple geographic locations, including Saudi Arabia, would be highly beneficial in future source-tracking, especially during epidemiological surveillance and outbreak investigations.

## Introduction

*Salmonella* is a highly diverse gram-negative anaerobic, facultative bacteria belonging to the family *Enterobacteriacea*e, and it is the most common foodborne pathogen worldwide. Contaminated food products of animal origin, such as poultry, eggs, and dairy products, play an important role in public health as they are considered the main source of *Salmonella* infections (salmonellosis). Although most salmonellosis cases are self-limited, yet it can be extremely life threating in cases involving patients at the extremes of age or those who are immunocompromised for whom appropriate antimicrobial therapy can be life-saving (Eng et al., 2015). According to the World Health Organization (WHO), *Salmonella* species are responsible for more than 93.8 million instances of gastroenteritis worldwide each year, along with more than 155,000 fatalities (Wu et al., 2021). Only a small number of research studies attempted to explore salmonellosis in Saudi Arabia. In one study, 5,202 salmonellosis cases were reported between 2013 and 2017 during a 5-year period (Alsayeqh, 2020). Another study revealed a consistent rise in salmonellosis cases, particularly during the summer and Hajj seasons, with chicken being the primary food source linked to these instances (Abd El Ghany et al., 2017). A few more investigations reported salmonellosis incidences in several Saudi Arabian provinces, including Asir, Al-Qatif, and Jeddah (Malik et al., 1993; Albreiki et al., 2004; Iyer et al., 2013).

Over the past decades, the increase of multidrug-resistant (MDR) forms of *Salmonella* in food-producing animals have progressively become a serious risk worldwide (Gymoese et al., 2019). This is likely due to the widespread and long-term use of common antimicrobials in poultry and animal husbandry for therapeutics, prophylaxis and growth promotion (Threlfall, 2002; Hughes and Heritage, 2004). Several reports described a strong correlation between antibiotic use in animals and the emergence of antibiotic resistance in foodborne bacteria associated with human disease (Levy et al., 1976; Holmberg et al., 1984; Fey et al., 2000; Hershberger et al., 2005; Marshall and Levy, 2011; Gaffga et al., 2012; Loharikar et al., 2013; Khoshbakht et al., 2018). This can happen directly by human contact with antibiotic-resistant bacteria from food animals, or indirectly from contact with resistant bacteria that have been spread to various components of the environment (e.g., water and soil) as a result of antibiotic use in food animals (Landers et al., 2012).

The poultry industry in Saudi Arabia has experienced major production growth in recent years. The increased demand for low-fat and high-protein diets among consumers has led to a remarkable increase in the consumption of poultry products in the country. Poultry is one of the most consumed animal proteins in Saudi Arabia, with an estimated average consumption of 47 kg of chicken meat and 120 eggs per year per person (Saudi Gazette, 2016). In 2020, the country produced 900,000 million tons of chicken meat, accounting for 60% of domestic consumption, and it is estimated to grow at a compound annual growth rate of 3.47% during 2020–2025 (Hussein, 2021). Due to the increased public demands for poultry products, the Saudi poultry industry has to overcome the occurrence of diseases and infections by following comprehensive animal husbandry practices and therapeutic applications, including the use of antimicrobial drugs and vaccinations. Although the application of antimicrobials has decreased the mortality and morbidity rates among commercial birds, yet the misuse of antimicrobials can result in treatment failures for animals and human as well. Unfortunately, research on chicken pathogens such as *Salmonella* is very limited in Saudi Arabia. Few studies examined the prevalence of certain pathogens without knowing the current phenotypic or genotypic patterns of antimicrobial resistance. According to these investigations, *S.* Enteritidis and *S*. Typhimurium are the two most common *Salmonella* serotypes in chicken meat (Al-Nakhli et al., 1999; Moussa et al., 2010; Archana et al., 2014; Badahdah and Aldagal, 2018). Therefore, this study aimed to characterize the genotypic and phenotypic antimicrobial resistance (AMR) profiles of *Salmonella enterica* isolates circulating in the Saudi poultry industry and explore phylogenomic diversity using WGS technology. The results of this study will improve our knowledge about the foodborne *Salmonella* current state in Saudi Arabia and provide valuable data about resistance and virulence mechanisms. This work also demonstrates the value of WGS technology as a promising tool for supporting evidence-based food safety hazard characterization for local policy decision-makers and human public authorities.

## Materials and methods

### Sample collection

A total of 39 *Salmonella* isolates were recovered from chilled chicken meat samples collected from local Saudi markets on a weekly basis in Riyadh city over a 3-month time period (May–July 2020), following the sampling guidelines of the document: “Integrated Surveillance of Antimicrobial Resistance in Foodborne Bacteria” by the World Health Organization (World Health Organization, 2017). Whole chickens were placed in a sterile plastic bags labeled and immediately transported in an ice-cooler to the Reference Laboratories of Microbiology at the Saudi Food and Drug Authority (SFDA).

### *Salmonella* isolation and identification

Isolation of *Salmonella* from chicken meat samples was carried out according to ISO 6579-1:2017/Amd 1:2020 (2020) (International Organization for Standardization, Geneva, Switzerland). Briefly, 25 g of chicken meat samples were dispensed in sterile plastic bags containing 225 ml of buffered peptone water (BPW, Oxoid, Hampshire, England, United Kingdom), then homogenized in a stomacher for 2 min and incubated at 37°C for 18 h. Then, 1 ml of BPW suspension was transferred into 10 ml of MULLER-KAUFFMANN Tetrathionate Novobiocin (MKTTn, Oxoid, Hampshire, England, United Kingdom) broth and incubated overnight at 37°C and 0.1 ml of BPW suspension was transferred into 10 ml of Rappaport Vassiliadis (RVS, Oxoid, Hampshire, England, United Kingdom) broth and incubated at 41.5°C for 24 h to eliminate other gram-negative bacteria. After incubation, a loopful of each broth was then streaked onto Xylose Lysine Desoxycholate agar (XLD, Oxoid, Hampshire, England, United Kingdom) which was incubated at 37°C for 24 h. Presumptive colonies with suspected *Salmonella* morphology were then selected, transferred into nutrient agar plates (Oxoid, Hampshire, England, United Kingdom), and incubated at 37°C for 24 h. After incubation, colonies were isolated and confirmed to the species level using matrix-assisted laser desorption/ionization time-of-flight (MALDI-TOF). All confirmed *Salmonella* isolates were also subjected to serological testing to determine their serotypes according to the Kauffman-White scheme by slide agglutination tests using commercially available mono- and poly-O groups *Salmonella* A, B, C, D, E antisera (Remel, Europe Ltd., United Kingdom). Isolates were further confirmed as *Salmonella* by real-time PCR 7500 using MicroSEQ™ *Salmonella* spp. detection Kit (Thermo Fisher, United States) in food. *Salmonella* isolates were then stored in the Biobank at −80°C at the SFDA.

### Antimicrobial susceptibility testing

Minimum inhibitory concentration (MIC) for all 39 *Salmonella* isolates was performed using Sensititer^™^ broth microdilution according to the manufacturer’s instructions (CMV3AGNF plates) (Thermo Fisher, Waltham, MA, United States) utilized by the National Antimicrobial Resistance Monitoring System (NARMS) (Karp et al., 2017). Each plate contains a total of 14 antimicrobial agents belonging to the following classes: (1) Beta-lactams: ampicillin, amoxicillin-clavulanic acid, cefoxitin, ceftiofur, and ceftriaxone; (2) Quinolones: nalidixic acid and ciprofloxacin; (3) Folic acid inhibitors: sulfisoxazole and trimethoprim-sulfamethoxazole; (4) Aminoglycosides: streptomycin and gentamicin; (5) Macrolides: azithromycin; (6) Phenicols: chloramphenicol; and (7) Tetracyclines: tetracycline. Results were interpreted according to the criteria of the Clinical and Laboratory Standards Institute (CLSI) guidelines (CLSI, 2019). Since there are no CLSI interpretive criteria for streptomycin, azithromycin, cefoxitin or ceftiofur for *Salmonella*, interpretive criteria defined by the National Antimicrobial Resistance Monitoring System were used (Gilbert et al., 2007). *Escherichia coli* ATCC 25922 was used as a quality control reference strain for the antimicrobial susceptibility tests.

### Whole-genome sequencing

Genomic DNA was extracted using the QIAamp DNA Mini kit following the manufacturer’s instructions (Qiagen, CA, United States) by a semi-automated machine (QIAcube). Genomic DNA purity was confirmed *via* an A260/A280 measurement (target ≥1.8) using QIAxpert (Qiagen, CA, United States), and DNA concentration was quantified using QFX Fluorometer (Denovix Inc., Delaware, United States) according to the manufacturers’ recommendations. Libraries were constructed using the Nextera XT DNA sample preparation and the Nextera XT Index Kits (Illumina, Inc., San Diego, CA, United States). After index PCR, samples were purified with 45 μl of Agencourt AMPure XP magnetic beads with a sample to beads ratio of 3:2 (Beckman Coulter, Brea, CA, United States), normalized by Qubit quantification and pooled to generate a 4 nM library. Paired-end sequencing was performed on the Illumina MiSeq platform, using 600-cycle MiSeq reagent kits (v3) with 5% PhiX control (Illumina Inc). Adapter trimming was performed automatically on the Illumina MiSeq during FASTQ generation. Raw data were demultiplexed using Illumina’s bcl2fastq tool (version 2.20) and checked for quality using Quast (version 4.6.3) (Gurevich et al., 2013). FastQ files were then uploaded to the Galaxy web platform, and *de novo* assembled at the public server at usegalaxy.org (Afgan et al., 2018) using Spades version 3.12.0 (Bankevich et al., 2012) with the default parameters.

### Bioinformatics analyses

Multi-locus sequence typing (MLST; version 2.0) was used to identify STs of the strains and the most common serovars (Larsen et al., 2012). Antimicrobial resistance genes (ARGs) were determined using ResFinder (version 4.1), where assembled contigs were screened for all the antibiotic drug classes in the database with nucleotide identity set at ≥90% nucleotide identity and ≥ 60% coverage (Bortolaia et al., 2020). Plasmid replicon typing was determined using PlasmidFinder (version 2.1) with a minimum percent identity at 95 and 60% for coverage (Carattoli et al., 2014). An additional validation step was performed to confirm the plasmid replicon types by comparing the finding with known replicon types from previously published complete plasmid sequences. Pathogenicity islands (PIs) were identified using SPIFinder (version 2.0), and settings were set at 90% as a minimum for identity and 60% for minimum query length. Virulence factors (VFs) were identified using the Virulence Factor Database^1^ with cutoff values of ≥90% identity and ≥ 50% coverage (Liu et al., 2019). Default parameters were used for all bioinformatic software tools unless otherwise specified.

### Genotype–phenotype correlation

Correlations between genotypic and phenotypic data obtained in this study were statistically evaluated by two-by-two table analysis (Mackinnon, 2000). ARGs presence/absence (obtained from WGS data) was compared with MIC phenotypic results representing the true resistance profile of the isolate. Intermediate phenotypes were not considered in this analysis. Accuracy, sensitivity, specificity, positive predicted value (PPV) and negative predicted value (NPV) were calculated as previously described (Parikh et al., 2008). Cohen’s kappa (κ) test was performed to measure the agreement of WGS to MIC in the identification of AMR (McHugh, 2012).

### Phylogenetic analysis

For genomic relatedness comparison between the 39 *Salmonella* isolates, FASTQ raw reads were uploaded to EnteroBase^2^ using the EneteroBase core genome (cgMLST) to determine the allelic profile of each genome assembly according to the 3,002- loci cgMLST scheme in *Salmonella* allelic profiles to construct minimum spanning tree (MST) through GrapeTree 1.5.0 (Zhou et al., 2018). We also investigated the hierarchical clustering (HierCC) designations for our collection of genomes implemented in the ‘cgMLST V2 + HierCCV1’ scheme in EnteroBase. The HierCC system assigns isolates to 13 different clusters defined by their resolution level and range from HC0 (no allelic differences) to HC2350 (genomes with up to 2,350 allelic differences) (Zhou et al., 2021). Here, the HierCC based on cgMLST with 50 allelic distances (HC50) was found to correspond to the four STs identified in this study and therefore was set as the threshold.

### Data availability statement

The raw nucleotide sequence reads generated in this study were submitted to the Short Read Archive (SRA) database of the National Center for Biotechnology Information (NCBI) under the BioProject accession number: PRJNA872154.

## Results

### Serotyping of *Salmonella* isolates

Among the 39 *Salmonella* isolates, four different *Salmonella* serovars were identified, including; *Salmonella* Minnesota (16/39), *Salmonella* Infantis (13/39), *Salmonella* Enteritidis (9/39), and one isolate was detected for *Salmonella* Kentucky (1/39).

### *In silico* MLST typing

Initial sequence analysis was performed using an *in silico* MLST approach based on information available at the *Salmonella enterica* MLST database (Alikhan et al., 2018) using seven loci (*aroC*, *dnaN*, *hemD*, *hisD*, *purE*, *sucA*, and *thrA*) to generate a sequence type (ST). Four distinct ST types were recognized, which corresponded to the four identified serovars. Among the 39 *Salmonella* isolates, 16 strains (41.1%) belonged to ST548 (*S.* Minnesota), followed by 13 strains (33.3%) belonged to ST32 (*S.* Infantis), nine strains (23.1%) belonged to ST11 (*S.* Enteritidis), and one strain (2.6%) belonged to ST198 (*S.* Kentucky).

### Antimicrobial susceptibility analysis

**Figure 1:**
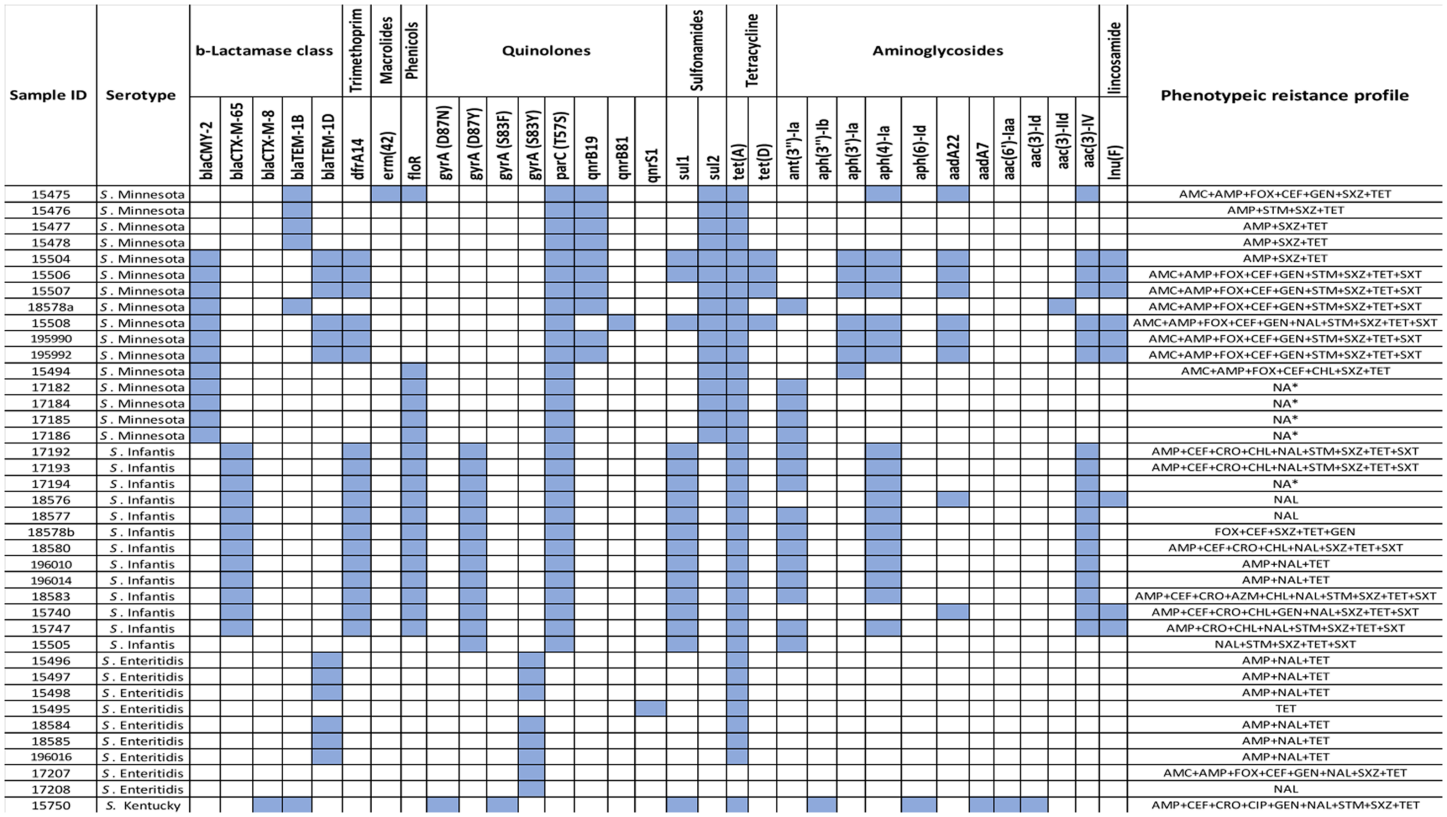
Heatmap of ARGs identified using ResFinder in 39 *Salmonella* isolates along with their phenotypic profiles.

Out of the 39 tested *Salmonella* isolates, four isolates with serotype Minnesota and one of serotype Infantis were eliminated from phenotype analysis due to their undetermined MIC values. Multidrug resistance, i.e., nonsusceptibility to at *least* one agent in *three* or more *antimicrobial categories*, was observed in 30/34 isolates (88%). The most common resistance phenotypes observed among *Salmonella* isolates were against tetracycline (91.2%, *n* = 31), ampicillin (82.4%, *n* = 28), sulfisoxazole 22 (64.7%, *n* = 22), nalidixic acid (61.8%, *n* = 21), azithromycin (41.2%, *n* = 14), trimethoprim/sulphonamides (38.2%, *n* = 13), gentamicin (32%, *n* = 11), and amoxicillin/clavulanic acid (29%, *n* = 10). Resistance to chloramphenicol and ceftriaxone was observed in 21% of the isolates (*n* = 7), and only one isolate (*S.* Kentucky 2.6%) was found to be resistant to ciprofloxacin. In general, there was no strong association between antimicrobial resistance phenotype with any particular serotype, except for *S*. Enteritidis isolates that showed susceptibility to most antibiotics compared to others (Figure 1). All *S.* Enteritidis isolates *were only* resistant to tetracycline, nalidixic acid, and ampicillin, with the exception of one isolate (ID: SA-17207). It was notable that the majority of *S*. Minnesota isolates displayed resistance to amoxicillin/clavulanic acid (66.6%) and gentamicin (53.8%), but were highly sensitive to nalidixic acid (8.3%) compared to other serotypes. Moreover, higher resistance rates toward ceftriaxone (50%) and chloramphenicol (50%) were observed among *S*. Infantis isolates compared to others. Details of antibiotic resistance results of the *Salmonella* serotypes are summarized in (Table 1).

**Table 1:** The antibiotic resistance patterns of *Salmonella* isolates to the tested antimicrobial agents from chicken meat samples.

Antibiotics	Resistance: Number (%)				
	*S.* Minnesota (*n* = 12)	*S.* Infantis (*n* = 12)	*S*. Enteritidis (*n* = 9)	*S*. Kentucky (*n* = 1)	Total (*n* = 34)
AMC	8 (66.6)	0 (0.0)	1 (11)	0 (0.0)	9 (26.5)
AMP	12 (100)	8 (66.6)	7 (77.7)	1 (100)	28 (82.4)
CRO	0 (0.0)	6 (50)	0 (0.0)	1 (100)	7 (20.6)
CHL	1 (8.3)	6 (50)	0 (0.0)	0 (0.0)	7 (20.6)
CIP	0 (0.0)	0 (0.0)	0 (0.0)	1 (100)	1 (2.9)
GEN	7 (53.8)	2 (16.6)	1 (11)	1 (100)	11 (32.4)
NAL	1 (8.3)	11 (91.6)	8 (88.8)	1 (100)	21 (61.8)
SXZ	12 (100)	8 (66.6)	1 (11)	1 (100)	22 (64.7)
TET	12 (100)	10 (83.3)	8 (88.8)	1 (100)	31 (91.2)
SXT	6 (50)	7 (58.3)	0 (0.0)	0 (0.0)	31 (38.2)
AZM	0 (0.0)	1 (8.3)	0 (0.0)	0 (0.0)	0 (0.0)
FOX	8 (66.6)	1 (8.3)	1 (11)	0 (0.0)	10 (29.4)
CEF	8 (66.6)	7 (58.3)	1 (11)	1 (100)	17 (50)
STM	7(53.8)	6 (50)	0 (0.0)	1 (100)	14 (41.2)

AMC, Amoxicillin/Clavulanic Acid; AMP, Ampicillin; FOX, Cefoxitin; CEF, Ceftiofur; CRO, Ceftriaxone; AZM, Azithromycin; CHL, Chloramphenicol; CIP, Ciprofloxacin; NAL, Nalidixic Acid; GEN, Gentamicin; STM, Streptomycin; TET, Tetracycline; SXZ, Sulfisoxazole; SXT, Trimethoprim/Sulphonamide.

### Detection of antimicrobial resistant genes (ARGs) using WGS

A total of 33 resistance genes conferring resistance to six categories of antimicrobials, including β-lactams, aminoglycosides, phenicols, tetracycline, trimethoprim, and sulfonamides, lincosamide, quinolones, and macrolides were identified located either chromosomally or on plasmids. Each genome of the 39 isolates harbor between 1 and 13 genes. All are described in detail below according to the different antimicrobial classes. *Aminoglycoside resistance genes*: Eleven distinct aminoglycoside resistance genes were detected including the three most common genes *aac (3)-IV*, *aph(4)-Ia*, and *ant(3″)-Ia* found in 19/39 (49%), 18/39 (46%), and 16/39 (41%) of our isolates, respectively. The gene; *aph(3′)-Ia* was identified exclusively in 7/39 (18%) of *S*. Minnesota isolates, whereas *aadA22* gene was found in 7/39 (23%) of *S*. Minnesota isolates, and 2/39 (5%) of S. Infantis isolates, respectively. Five genes, *aph(3″)-Ib*, *aph(6)-Id*, *aadA7*, *aac(6′)-Iaa*, and *aac(3)-Id* were exclusively found in 1/39 (3%) *S*. Kentucky isolate. One gene, *aac (3)-IId*, was found in only 1/39 (3%) *S*. Minnesota isolates. *Beta-lactam resistance genes*: Five distinct ß -lactam resistance genes were detected, including: *Bla*_CMY-2_ was found only in 12/39 *S.* Minnesota (31%) isolates; *bla*_CTX-M-65_ was detected only in 12/39 *S. Infantis* (31%) isolates; *bla*_TEM-1D_ was found in 6/39 *S.* Enteritidis (15.4%) and 6/39 *S.* Minnesota (15.4%) isolates; *bla*_TEM-1B_ was detected in 5/39 S. Minnesota (13%); 1/39 *S.* Kentucky (3%) isolates; and *bla*_CTX-M-8_ was found only in 1/39 *S.* Kentucky (3%) isolate. *Quinolone resistance genes*: Resistance to fluoroquinolones (ciprofloxacin and nalidixic acid) was mainly associated with either chromosomal mutations on *parC* and *gyrA* genes or plasmid mobilization of *qnr.* All *S.* Minnesota and *S.* Infantis isolates were found to have *parC* (T57S), where *gyrA* mutation (D87Y) was exclusively found in *S.* Infantis. The *gyrA* mutation (S83Y) was carried out only by *S.* Enteritidis isolates. One isolate, *S.* Kentucky, had two *gyrA* mutations (D87N and S83F). The plasmid-mediated quinolones *qnrB19* gene was detected in 10/39 *S*. Minnesota isolates, whereas *qnrB81* and *qnrS1* genes were detected only once in 1/39 *S*. Minnesota and 1/39 *S*. Enteritidis, respectively. *Tetracycline resistance genes*: Only two tetracycline resistance genes were detected, including the most common *tetA* gene found in 37/39 (95%) isolates which corresponded with the high levels of reduced susceptibility to tetracycline detected by MIC, where *tetD* was found in 4/39 (10%) *S*. Minnesota isolates. *Antifolate resistance genes*: dihydropteroate synthase gene *sul1* was the most abundant gene among our isolates except in *S*. Enteritidis, whereas *sul2* gene was only found among *S*. Minnesota Isolates. One resistance gene for trimethoprim (*dfrA14*) was detected in 12/39 (30.7%) of *S*. Infantis, and 6/39 (15.4%) of *S*. Minnesota isolates. *Phenicol and Macrolide*: one resistance gene for phenicol and macrolide was detected in our isolates: *floR* and *erm(42)*, respectively. The phenicol resistance gene (*florR*) was detected in 12/39 (30.7%) of *S.* Infantis, *and* 6/39 (15.4%) of *S.* Minnesota isolates. The macrolide-resistant gene *erm(42)* was detected in only one *S*. Minnesota. Interestingly, genomic AMR profiling identified the presence of two additional resistance genes, *lnu(F)* and *mcr-9*, that were not phenotypically tested. The distribution of antimicrobial resistance genes in all 39 isolates, along with their phenotypic profiles, are shown in (Figure 1).

### Genotypic-phenotypic correlation

A total of 34/39 isolates were tested for genotype–phenotype correlation due to the absence of phenotypic testing data for five isolates. Genotypes predicted phenotypes varied in sensitivity (100–93.8%) and in specificity (100–33.3%) among tested isolates. The sensitivity was highest (100%) for resistance against phenicol and fluoroquinolones, and lowest for aminoglycosides (93.8%). On the other hand, the highest specificity was (97%) for resistance against macrolides, and lowest was for tetracycline (33.3%) (Table 2). The overall kappa score (*κ* = 0.82) indicated that WGS gene presence was acceptably predictive of susceptibility testing and showed “strong” agreement with phenotypic data. Two antimicrobial classes (ß-lactam and macrolides) showed almost perfect agreement (*κ* > 0.90) between phenotype and genotype, three antimicrobials (Sulfonamides, tetracycline and phenicol) showed strong agreement (*κ* = 0.80–0.90), where aminoglycosides and fluoroquinolones showed moderate agreement (*κ* = 0.60–0.79) between genotype and phenotype. The weakest phenotype–genotype correlation was mostly pronounced by the presence of fluoroquinolone chromosomal mutation *parC (T57S)* in 13 isolates which were phenotypically susceptible. No perfect agreement was observed among our tested isolates where some isolates had resistant phenotype with no genetic explanation or vice versa where ARGs were detected in some isolates with susceptible phenotype. Detailed information on the agreement between ARGs identified by ResFinder database and phenotypic profile determined by MIC values are shown in (Table 2).

**Table 2:** Genotype–phenotype correlation between 34 *Salmonella* strains isolated from chicken meat.

Antimicrobials	Phenotype: R		Phenotype: S		Sensitivity %	Specificity %	Accuracy %	Kappa*
	genotype: R (TP)	genotype: S (FP)	genotype: R (FN)	genotype: S (TN)				
Fluoro(quinolones)	21	13	0	0	100	DBZ*	61.7	0.60
Beta-lactams/beta-lactam inhibitors	28	2	1	3	97	60	91.2	0.90
Tetracycline	30	2	1	1	96.7	33.3	91.2	0.89
Sulfonamides	21	4	1	8	95.5	66.6	85.3	0.84
Phenicols	7	6	0	21	100.0	77.7	82.4	0.81
Macrolides	0	1	1	32	DBZ*	97	94.1	0.92
Aminoglycosides	15	7	1	11	93.8	61	76.5	0.76

R, Resistant; S, Susceptible; True positive (TP), ARG present and MIC resistant; False positive (FP), ARG present and MIC susceptible; False negative (FN), ARG absent and MIC resistant; True negative (TN), ARG absent and MIC susceptible.

*Division by Zero (DBZ): values were not able to calculate when genotype resistance was absent, or phenotype resistance was not observed due to division by zero.

*Kappa correlation (κ): ≥0.90 (almost perfect agreement), 0.80–0.90 (strong agreement), 0.60–0.79 (moderate agreement), 0.40–0.59 (weak agreement), 0.20–0.39 (minimal agreement), and 0.0–0.20 (no agreement).

### Detection and typing of plasmids using WGS

**Figure 2:**
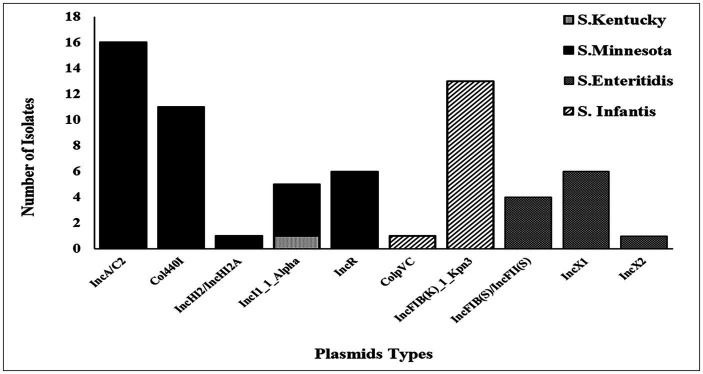
Plasmids distribution among 39 *Salmonella* isolates.

The accuracy and reliability of the *in silico* plasmid typing results were validated by comparing the results with known replicon types from previously published full plasmid sequences. Our results identify 11 distinct plasmid replicon types among the 39 genomes analyzed in this study, including IncA/C2 (reference accession no: CP080515.1), IncFIB(K)_1_Kpn3 (CP052818.1), Col440I_1(CP060511.1), IncR (OW968256.1), IncX1 (CP091083.1), IncI1_1_Alpha (MW590592.1), IncFIB(S)/IncFII(S) (CP050711.1), IncHI2/IncHI2A (CP080514.1), IncX2 (CP091083.1), and ColpVC (CP082746.1). The findings revealed that there was a variation in the replicon types identified among serotypes (Figure 2). Thirty-seven (37/39) of these strains were positive for at least one plasmid. Five plasmids: IncA/C2, Col440I_1, IncHI2/IncHI2A, and IncR, were exclusively found in ST548; *S.* Minnesota, with IncA/C2 being the most frequent plasmid. All isolates belonging to ST32; *S.* Infantis carried one replicon type IncFIB(K)_1_Kpn3 plasmid, except one isolate (ID: SA-18583), which carried IncFIB(K)_1_Kpn3 and ColpVC plasmid. Isolates belonging to ST11; *S.* Enteritidis serotype carried IncFIB(S)/IncFII(S), IncX1 and IncX2 with different frequencies. Multi-drug resistance IncI1_1_Alpha replicon type was found only in two isolates belonging to ST198, *S.* Kentucky and ST548, *S.* Minnesota (Figure 2).

### Phylogenetic relationship of *Salmonella* isolates

**Figure 3:**
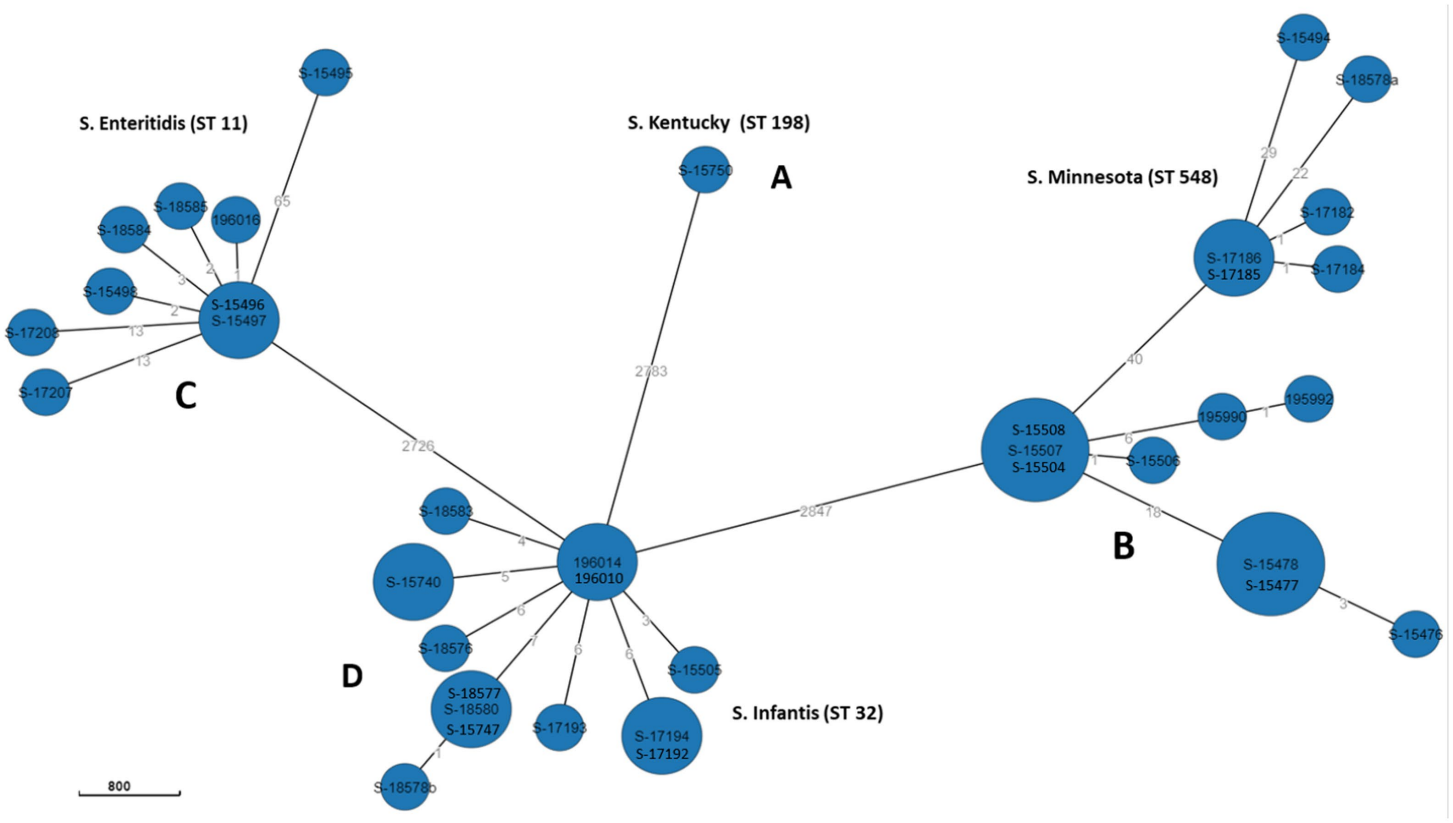
GrapeTree MST based on cgMLST data.

The relatedness between samples from this study was determined by constructing MST using “cgMLST V2” algorithm. In Enterobase, the number of core loci included in the cgMLST scheme for *Salmonella* is 3,002 gene. The differences in the nucleotide sequences of these loci determine the clustering of the 39 isolates. Separation of isolates by hierarchical clustering system corresponding with their STs was set using HierCC HC50, which diversify the 39 genomes into four phylogroups (A, B, C, and D) consisting with their STs assignment; group A: *S.* Minnesota ST548; group B: *S.* Infantis ST32, grope C: *S.* Enteritidis ST11, and group D: *S.* Kentucky ST198. This means that CgMLST diversity of strains with the same ST varied from 0 to 50 allelic differences. However, one *S.* Enteritidis isolate (ID: S-15495) was found to have 65 allelic differences and therefore did not cluster within the same group (Figure 3). Overall, large phylogenetic differences were observed between the four phylogroups in their core genome, where ST32 and ST11 were found to be closer with an average of 2,726 allelic differences, and both were distant from the other strains belonging to ST198 and ST548 with an average of 2,783 and 2,847 allelic differences, respectively (Figure 3).

### Detection of PIs and virulence factors using WGS

**Figure 4:**
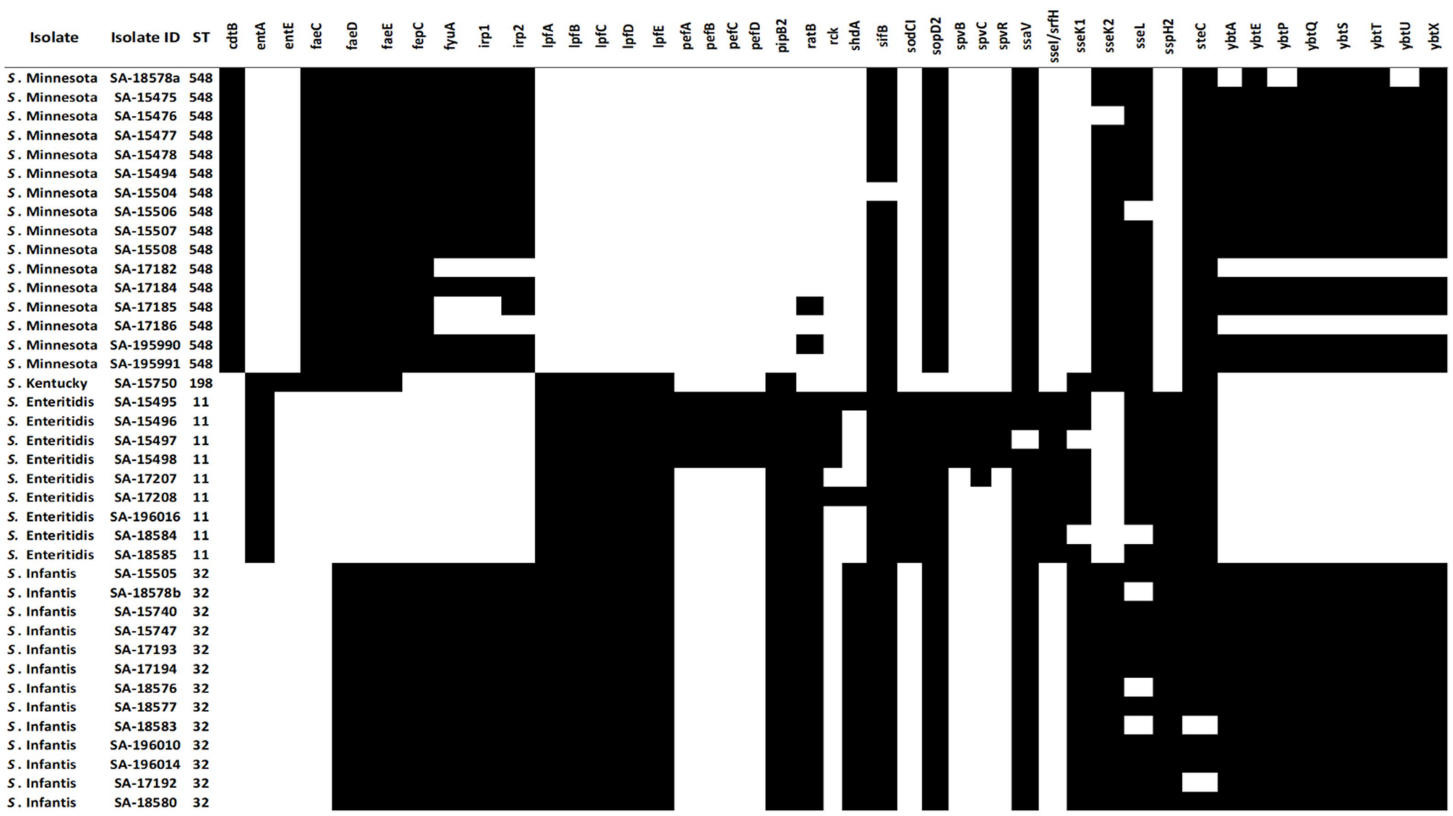
Distribution of variable virulence factors in the genomes of 39 *Salmonella* isolates. Black cells indicate the presence of virulence factors in salmonella isolates, and white cells indicate the absence of virulence genes.

To understand the pathogenicity repertoire of *Salmonella* isolates, genetic virulence factors were queried in the 39 genomes. All *Salmonella* genomes were analyzed for virulence factors using SPIFinder 1.0.^3^ One pathogenic island (PI), the centisome 63 (C63PI), was conserved among all 39 genomes, PIs including SPI-14 (SPI-14.SGC-8, SPI-14.SGA-8), and SPI-13 (SPI-13.SGD-3, SPI-13.SGG-1, and SPI-13.SGA-10) were detected in all isolates with the exception of *S.* Kentucky isolate. One of each SPI-3, SPI-4, SPI-2, SPI-5, and SPI-1 were detected in 33 (85%), 28 (72%), 24 (62%), 19 (42%), and 13 (33%) isolates, respectively. To identify key pathogenicity genes of *salmonella* isolates, we investigated the distribution of virulence genes. To do this, all 39 *salmonella* genomes were locally aligned against the Virulence Factors Database (Liu et al., 2019). The complete virulence gene profiles of each *Salmonella* isolate are shown in (Figure 4). Identified virulence factors were collectively grouped under nine categories including genes involved with the adhesion, invasion, colonization, secretion, toxicity, serum resistance, survival, magnesium, and iron uptake. The similarity rate varied between 80 and 100% of identity for all strains, with coverage between 85 and 100%. The majority of the virulence genes (87/131) were conserved among all isolates, mostly belonging to type III secretion system (T3SS) encoded by *Salmonella* SPI-1 and -SPI-2, followed by several other gene clusters, including curli fimbrial adherence, colonization, iron and magnesium uptakes, antimicrobial resistant, and flagellar apparatus biosynthesis determinants. On the other hand, the detection rate of the remaining 44 virulence genes was variable (Figure 4). For example, genes encoding for the virulent yersiniabactin operon (*fyu*A, *irp*12, *ybt*AEPQSTUX) were found to be predominant in *S.* Minnesota, and *S.* Infantis isolates. Additionally, the Typhoidal toxin; Cytolethal distending toxin gene (*ctdB*) was detected solely within the *S.* Minnesota isolates, and the iron uptake (*entE*) gene was exclusively found in *S.* Kentucky. Notably, a cluster of genes was missing in *S.* Minnesota isolates and found variably in other serotypes, including fimbrial adhesions (*lpfABCDE and pefABCD*), enterotoxins, T3SS effectors (*spvBRC*), iron uptakes (*entAE*), and resistant to complement killing gene (*rck*). The phage carrying the periplasmic superoxide dismutase gene, *sodCI*, was only detected in *S.* Enteritidis isolates which may contribute to their virulence. The remaining virulence factors were variably shared between all *S.* Enterica serotypes, where strains belonging to the same serovar exhibited a distinct virulence profile (Figure 4).

## Discussion

Lately, increasing rates of MDR *Salmonella* are a significant concern in poultry production that needs to be monitored continuously. With few exceptions, most of the tested strains in our study exhibited MDR phenotypes and genotypes, confirming the acquisition of AMR determinants among *salmonella* strains. Moreover, our results confirmed previous observations of the widespread resistance to older and conventional antimicrobial agents (McDermott et al., 2018), where we saw a high level of resistance (≥ 50%) among the tested isolates against tetracycline, ampicillin, sulfisoxazole, and nalidixic acid. These findings are comparable to data from other studies among *Salmonella* isolates recovered from poultry in other countries (Zhu et al., 2017; Hossain et al., 2021; Osman et al., 2021; Wei et al., 2021), which may indicate the extensive use of these antimicrobial drugs in the poultry industry for rapid growth and disease prevention. In contrast, azithromycin and ciprofloxacin resistance were rare, with only one *S*. Infantis and one *S*. Kentucky showing resistance phenotype against these two antibiotics, respectively.

Different determinants conferring resistance against different antibiotics were found in our sequenced strains, with each isolate harbored between 1 to 13 ARGs with an average of 7.7 genes. Moreover, different genetic antimicrobial resistance patterns were observed among the tested isolates, where *S*. Enteritidis isolates had the lowest ARGs presence compared to the other serotypes (Figure 1). Interestingly, several detected ARGs were frequently found residing on mobile elements such as plasmids which are responsible for the widespread dissemination of antimicrobial resistance in nature (Van Hoek et al., 2011). For example, all *S.* Infantis isolates were positive for IncFIB(K)_1_Kpn3 plasmid that carries *bla*_CTX-M-65_ gene, which confers resistance to β-lactams. IncFIB(K)_1_Kpn3 plasmid has contributed to the rise of *S.* Infantis as one of the dominant serotypes in poultry in Europe and the United States, where the prevalence of this serotype increased up to 70% in the past few years (García-Soto et al., 2020). Moreover, *S.* Infantis carrying *bla*_CTX-M-65_ has emerged lately in poultry and caused human infections around the world (Aviv et al., 2014; Brown et al., 2018). Another observation was noticed where all *S.* Minnesota shared resistance to sulfonamides, tetracyclines and β-lactams conferred by *sul2, tet(A)* and *bla_CMY-2_* resistant genes, which were carried on IncA/C_2_ plasmid (formerly known as IncC). Interestingly, similar recent findings were observed in Brazil and in meat products imported from Brazil into the UK, demonstrating the association of *sul2*, *tetA* and *bla*_CMY-2_ genes with IncC plasmids in *S*. Minnesota isolates (Alikhan et al., 2022). In general, *I*ncA/C_2_ plasmid has been reported and characterized as a major contributor to MDR in *Salmonella* in several countries, mainly through imported chicken meat from Brazil (Heider et al., 2009; Glenn et al., 2013; Campos et al., 2018; Rozwandowicz et al., 2018; Van den Berg et al., 2019; Kipper et al., 2020; Alikhan et al., 2022). Therefore, exploring the link between the poultry raised in Brazil, in particular, and other countries with Saudi Arabia is necessary to determine the genetic relatedness of *S*. Minnesota isolates.

In the current study, we compared the antibiotic resistance phenotypes with their corresponding genotypes to determine the utility of WGS results in predicting phenotypic drug susceptibility to different antibiotics. β-lactam and macrolides antibiotic classes showed the highest correlation between phenotypes and genotypes among tested isolates (*κ* > 0.90), followed by sulfonamides, tetracycline and phenicols (*κ* = 0.80–0.90), suggesting that ARGs based WGS data could be used as an acceptable predictor of AMR phenotype for these compounds. In contrast, a moderate agreement was observed for two antibiotic classes: aminoglycosides and quinolones (*κ* = 0.60–0.79), leading to an accuracy of 76.5 and 61.7%, respectively, indicating a slightly high discrepancy in phenotype–genotype correlations. Based on our results, most observed discrepancies were due to false positive results, i.e., isolates that are phenotypically susceptible but have an associated genotype. False positives may be caused by the detection of silenced antibiotic resistance genes that do not express resistance phenotype as reported previously in *Salmonella enterica* (Neuert et al., 2018; Kime et al., 2019; Kuijpers et al., 2019; McMillan et al., 2019; Vk et al., 2019; Matchawe et al., 2022). A few numbers of isolates showed false negative results, i.e., isolates that were phenotypically resistant but genotypically susceptible. This is most likely due to the presence of new ARG variants that have not been discovered yet and, therefore, included in the reference database used for prediction (Neuert et al., 2018; Maunsell et al., 2021). Another reason for the observed false negative results would be the existence of unknown mechanisms of resistance, such as the expression of efflux pumps and cell wall permeability (Webber and Piddock, 2003; Leon et al., 2018; Maunsell et al., 2021). No perfect agreement was established between all phenotypically confirmed *Salmonella* isolates and *in silico* predicted ARGs. Future research should incorporate additional comparisons among various ARGs detection systems, such as CARD database system or AMRFinder tool, for further analysis of discrepant results with a larger number of tested isolates (McArthur et al., 2013; Feldgarden et al., 2019). Removal of isolates with three or more false positive/negative results across three or more antibiotic classes is another strategy that may be used to exclude isolates that may have potential discrepancies, testing errors, or other confounding factors (Feldgarden et al., 2019).

*Salmonella* virulence factors aid in pathogenicity and host colonization by assisting the pathogen in attaching to, invading, and replicating within host cells and avoiding host defenses using different mechanisms such as adhesion systems, capsule, flagella, and toxins (Jajere, 2019). Virulence factors are frequently clustered together in PIs, which are often found on mobile genetic elements such as plasmids (Cheng et al., 2019). Eleven *Salmonella* SPIs (C63PI, SPI-1, SPI-2, SPI-3, SPI-4, SPI-5, SPI-14. SGC-8, SPI-14. SGA-8, SPI-13SGD-3, SPI-13.SGG-1, SPI-13. SGA-10), and 131 virulence factors were commonly found among tested *Salmonella* isolates. Out of the 11 identified SPIs, SPI-1, SPI-2, SPI-3, SPI-4, and SPI-5 play a more critical role in *Salmonella* pathogenicity. In contrast, SPI-13 and SPI-14 are associated more with the regulation of SPI gene expression or other associated effector proteins (Matchawe et al., 2022). The C63PI was detected in all tested isolates, which may explain its role in *Salmonella* survival during iron uptake, hence its conservation among all *Salmonella* strains. About 66.4% of the virulence factors (87/131) were conserved among all *Salmonella* isolates, including T3SS genes located within the *Salmonella* SPI-I and SPI-2, where SPI-I is mainly involved in the infection initiation stage, and SPI-2 is required for systemic infection (Matchawe et al., 2022). These factors are part of the core genes with an essential function for *Salmonella,* such as infectivity, transmission, colonization, and survival (Pornsukarom et al., 2018). On the other hand, 33.6% of virulence determinants (44/131) were highly diverse or confined to a single serovar indicating variation in pathogenicity among *Salmonella* serovars. For example, the plasmid replicon IncFII(S)/IncFIB(S) virulence-associated genes; the *pef* operon comprising *pefABCD*, the *spvBCR*, and the *rck* gene were all solely identified in *S*. Enteredites. On the contrary, all *S*. Minnesota isolates were found exclusively to harbor *cdtB* gene, which is a toxin that was originally thought to be restricted to serovars Typhi and Paratyphi A, that causes cell arrest due to DNA damage leading to typhoid fever in humans (Tamamura et al., 2017; Chang et al., 2019; Rhen, 2019). At present, the role of this effector in *S.* Minnesota remains poorly characterized, and there is no evidence that the presence of *cdtB* gene is related to typhoid fever or increased virulence of the strain. One gene cluster encodes for yersiniabactin-mediated iron acquisition system detected in both *S.* Minnesota, and *S.* Infantas isolates. According to previous studies, the presence of this gene cluster normally results in increase growth in the infection site, biofilm formation (Hancock et al., 2008) and an increase in the overall pathogenicity in chicken (Tu et al., 2016). This gene cluster is predicted to present in a chromosomal region called the highly pathogenic island (HPI) and is usually associated with *Escherichia coli* (Carniel, 1999). Therefore, further studies on molecular analysis of the gene content of SPIs in different *Salmonella* strains could help to enhance our understanding of *Salmonella* pathogenicity.

To the best of our knowledge, this is the first study that employs WGS for AMR characterization of *Salmonella* isolates from chicken meat samples in Saudi Arabia, yet it has some limitations. First, this study was conducted at Riyadh, a single city in Saudi Arabia. Therefore, our findings may not be generalized to other geographical regions in the country. Second, the low number of tested chicken samples does not allow for generalized prevalence conclusions; therefore, future studies should include a larger sample size. Third, tested samples were collected from different brands (results not shown), and the proportion of each brand in the total sample was not evaluated. It is expected that different brands are under different kinds of management programs and that there are different risks regarding the prevalence of *Salmonella* spp. and the use of antimicrobials. Hence, future studies should focus on investigating poultry companies along to their management programs. Finally, information regarding the drug use in the poultry industry is nonexistent and needs to be present in Saudi Arabia. The absence of such information prevents us from fully determining the effect of subtle changes in the level of use of various antibiotics on resistance among bacteria recovered from chickens. Therefore, multidisciplinary efforts are needed to limit the usage of some antibiotics that showed high resistance patterns in Saudi poultry production. New measures should immediately be applied that would limit the use of some of the highly resistant drugs in animals and ultimately help to reduce the selection pressures that generate antimicrobial resistance. Suitable alternatives to the extensive use of antibiotic drugs can also be implemented, such as vaccination and alterations in poultry management. Other changes could also be applied, such as targeted use of antimicrobials with a more limited dosage and duration to avoid selection for resistance to critical human therapeutics.

## Conclusion

In conclusion, this study reveals the high prevalence of MDR *Salmonella* in chicken meat and underscores the need for rigorous surveillance of AMR in food production animals in Saudi Arabia. WGS technology enabled us to obtain a comprehensive resistome profile that can be useful in developing AMR control programs for reducing the burden of ARGs circulating through the food chain. Additionally, this study yields important information on *Salmonella* populations from poultry based on cgMLST, resolving the relationship among circulating strains. Moreover, this study showed an acceptable concordance between phenotypic antimicrobial susceptibility and predicted genotypes from WGS results, suggesting that this technology could be used in combination with phenotypic tests for surveillance purposes. Finally, information derived from this study can be used as a valuable reference for future investigations and to inform public health authorities aimed at limiting further dissemination of ARGs and hence aid in the fight against the global public health AMR threat.

## Data availability statement

The datasets presented in this study can be found in online repositories. The names of the repository/repositories and accession number(s) can be found in the article/Supplementary material.

## Author contributions

SA conceived and designed the study. MM, FA-R, AAA, MSA, EA, MJA, SA-A, MA-H, AMA, and ATA performed the sampling and the experiments. KA, EA, ASA, and LM analyzed the data. KA, EA, and ASA wrote the original draft. KA, LM, MAA, SA, and MFA reviewed and edited the manuscript. All authors contributed to the article and approved the submitted version.

## Funding

This work was funded by Saudi Food and Drug Authority (SFDA) in Riyadh, Saudi Arabia.

## Conflict of interest

The authors declare that the research was conducted in the absence of any commercial or financial relationships that could be construed as a potential conflict of interest.

## Publisher’s note

All claims expressed in this article are solely those of the authors and do not necessarily represent those of their affiliated organizations, or those of the publisher, the editors and the reviewers. Any product that may be evaluated in this article, or claim that may be made by its manufacturer, is not guaranteed or endorsed by the publisher.

## Footnotes

^1^ http://www.mgc.ac.cn/VFs/main.htm

^2^ https://enterobase.warwick.ac.uk

^3^ https://cge.cbs.dtu.dk/services/SPIFinder/
